# The distinct effects of orally administered *Lactobacillus rhamnosus* GG and *Lactococcus lactis* subsp. *lactis* C59 on gene expression in the murine small intestine

**DOI:** 10.1371/journal.pone.0188985

**Published:** 2017-12-08

**Authors:** Chise Suzuki, Ayako Aoki-Yoshida, Reiji Aoki, Keisuke Sasaki, Yoshiharu Takayama, Koko Mizumachi

**Affiliations:** Institute of Livestock and Grassland Science, National Agriculture and Food Research Organization (NARO), Tsukuba, Ibaraki, Japan; The University of Tokyo, JAPAN

## Abstract

The molecular mechanisms of strain-specific probiotic effects and the impact of the oral administration of probiotic strains on the host’s gene expression are not yet well understood. The aim of this study was to investigate the strain-specific effects of probiotic strain intake on gene expression in the murine small intestine. Two distinct strains of lactic acid bacteria, *Lactobacillus rhamnosu*s GG (GG) and *Lactococcus lactis* subsp. *lactis* C59 (C59), were orally administered to BALB/c mice, daily for 2 weeks. The total RNA was isolated from the upper (including the duodenum) and lower (the terminal ileum) small intestine, and gene expression was assessed by microarray analysis. The data revealed (1) oral administration of C59 and GG markedly down-regulated the expression of genes encoding fibrinogen subunits and plasminogen in the upper small intestine; (2) administration of more than 1 × 10^7^ CFU/day of GG changed the gene expression of the host ileum. (3) strain- and dose-related effects on various GO biological processes; and (4) enrichment for B cell-related Gene Ontology terms among up-regulated genes in the terminal ileum of mice administered the 1 × 10^9^ CFU/day of GG. The distinct effects of GG and C59 on gene expression in the intact small intestine provide clues to understand how the health beneficial effects of specific strains of probiotic bacteria are mediated by interactions with intestinal cells.

## Introduction

Probiotics are defined as “live microorganisms, which, when administered in adequate amounts, confer a health benefit on the host” [1]. Various strains of lactic acid bacteria (LAB) and bifidobacteria are utilized as probiotics and exert strain-specific beneficial effects on health by regulating the gut ecosystem, preventing pathogen adherence, and interacting with gut associated lymphoid tissue [2, 3]. The beneficial health effects of probiotics partially depend on the components of the bacterial cell, such as peptidoglycan, lipoteichoic acids, and bacterial DNA, known as microbe-/pathogen-associated molecular patterns (MAMPs/PAMPs) [4]. Interaction between Toll-like receptors (TLRs) and those bacterial components, as TLR ligands, are thought to mediate the interaction between the host’s epithelial immune cells and probiotic cells [4, 5]. Metabolites produced by probiotics also contribute to their beneficial health effects. For example, acetate produced by bifidobacteria modulates the intestinal defense responses of the host and protects epithelial cells from infection [6]. Furthermore, butyrate produced by the microbiota in the large bowel induces the differentiation of colonic Treg cells in mouse [7]. Animal models of various diseases or samples from patients [8, 9] are used to investigate the effects of probiotics, but the intestinal responses of healthy animals to probiotics have not been investigated. In addition, although many studies have reported the beneficial effects of probiotics, there have been few comparative studies of probiotics. Further, since the number of indigenous microbiota in the intestine is estimated to be 10^12−14^ cells, it is unclear how probiotics could have a beneficial effect when the ratio of intestinal bacteria to probiotic bacteria may be as low as 1:100,000 after probiotic administration. However, supplemented probiotics may manifest their effects on intestinal microbiota via the host's immunity. The host immune system may react to the administered LAB and mount specific responses to strain-specific components. The lack of an appropriate control LAB strain has made it difficult to compare the effects of different probiotics. Conventionally, a probiotic strain and a vehicle control are used for oral administration, but a vehicle control is only rarely satisfactory. In this study, we tried to overcome this difficulty by studying the strain-specific effects of two orally administered LAB strains.

*Lactobacillus rhamnosus* GG (GG) is one of the most extensively studied probiotic strains. GG was originally isolated from fecal samples of a healthy human adult [10] and is resistant to bactericidal agents in the intestinal environment, such as high gastric acid and bile concentrations, and can adhere to the intestinal epithelial layer via pili [11–13]. The beneficial effects of GG intake on the gut microbiota have also been investigated [14, 15]. GG inhibits the adherence of pathogens to the intestinal epitherium, enhances the epithelial barrier function, and modulates host immune responses [16, 17]. Neonatal colonization of mouse by GG promotes intestinal development and protects the intestine from inflammation, maintaining the host’s health [18]. In various disease models, such as allergic diseases [19], and colitis and obesity [20], administration of *Lactobacillus* GG alleviates the symptoms of disease [11, 21]. Furthermore, randomized trials demonstrated beneficial effects of GG ingestion on atopic disease [22, 23], nosocomial gastrointestinal infections [24], pediatric obesity-related liver disease [25], and acute gastroenteritis in children [26].

*Lactococcus lactis* subsp. *lactis* C59 was isolated from cheese at the Institute of Livestock and Glassland Science, NARO [27]. Contrary to the strong immunostimulatory activity of GG, the immunostimulatory activity of C59 is very low. Its ability to stimulate a macrophage-like cell line J774.1 to induce interleukin (IL) 12 and interferon (IFN) γ was the weakest from a panel of 10 *Lactobacillus* and *Lactococcus* strains [28]. Upon the administration of live C59, the strain suppresses the increase of serum IgE antibody levels in ovalbumin (OVA)-sensitized mouse [28]. *Ex vivo* experiments revealed that the inhibition of IgE antibody production by C59 is caused by the suppression of IL-4 production [28], in a manner different from strains that enhance the Th1 response. By contrast to the adherence of GG to the intestinal epithelial layer, C59 is excreted live in feces within a day of administration, suggesting that it does not adhere to intestinal cells [28].

Various MAMPs implicated in the beneficial effects of GG, such as immunostimulatory oligodeoxynucleotides in the GG genomic DNA [29], the pili [30], lipoteichoic acid [31, 32], and surface proteins [33], have been investigated. The stimulation of *Tlr3* gene expression by GG is observed both *ex vivo* and *in vivo* [34]. TLR3 is a nucleotide-sensing TLR, which is activated by double-stranded RNA (dsRNA). LAB contain more dsRNA than pathogenic bacteria and trigger IFN-β production [35]. The GG cell surface contains high molecular weight, galactose-rich heteropolymeric exopolysaccharide (EPS) molecules, which may negatively impact the adherence of GG, possibly by shielding the adhesins [36]. On the other hand, C59 harbors unique EPS consisting of glucose, *N*-acetylglucosamine, and rhamnose, although most *L*. *lactis* EPS molecules analyzed thus far contain galactose [37]. An *in vitro* experiment showed that C59 may activate plasminogen; it converts plasminogen to plasmin in a reaction catalyzed by a serine protease [38]. Thus, GG and C59 have distinct characteristics.

The aim of this study was to investigate the effect of oral administration of LAB strains C59 and GG on the gene expression in the murine small intestine, i.e., the first site of probiotic interaction with the host’s intestinal cells. By using two distinct LAB strains, we expected to detect common responses and administered strain-specific responses. Comparison of the effect of these distinct strains on the gene expression in the small intestine could help elucidate the distinct responses of intestinal cells and the strain-specific mechanism of probiotics.

## Materials and methods

### Bacterial strains

Strain GG (ATCC 53103) was kindly provided by Takanashi Milk Products Co. Ltd. GG was cultured in Difco lactobacilli de Man, Rogosa and Sharpe broth (MRS) (Becton Dickinson, Sparks, MD) for 18 h at 37°C. Strain C59 was isolated from cheese [27] and is maintained in the LAB collection at NILGS (Institute of Livestock and Glassland Science, NARO). C59 was grown in Difco M17 broth (Becton Dickinson) containing 0.5% glucose as a carbon source (GM17) for 18 h at 30°C. Bacterial cells were harvested by centrifugation at 6000 × *g* for 15 min at 4°C, and washed twice with phosphate-buffered saline (PBS); bacterial cell suspensions (OD_620_ = 10) were prepared in PBS. The suspensions were divided into small aliquots and stored at -80°C until administration.

### Animal experiments

All animal care and use protocols were implemented in accordance with the animal experimentation guidelines of the National Agriculture and Food Research Organization (NARO; Ibaraki, Japan). The study protocol was approved by the Animal Care Committee at the National Institute of Livestock and Grassland Science (Ibaraki, Japan; permit numbers: 07062806, 08032651, and 09032637).

Six-week-old male BALB/c (Balb/cAnNcrl) mice (*n* = 90; average starting weight, 23.0 g) were purchased from Charles River Japan, Inc. (Kanagawa, Japan) and acclimatized for 1 week before the start of experiments. Three animals were housed per cage under a 12 h light/dark cycle and allowed free access to water. The basic feed was a nutritionally complete diet (AIN-93G, Oriental Yeast, Tokyo, Japan). The mice had *ad libitum* access to water and the diet. In each experiment, mice in the control group received 0.2 ml of phosphate buffer saline (PBS) and mice in the LAB administered groups received 0.2 ml of bacterial suspension in PBS, once per day for 15 days, using an intragastric stainless steel feeding tube. The final LAB administration was followed by a 3 h fast to make the period from the last feed equal. Then, the mice were sacrificed by cervical dislocation under pentobarbital anesthesia (5%, 75 μl/mouse).

**Fig 1:**
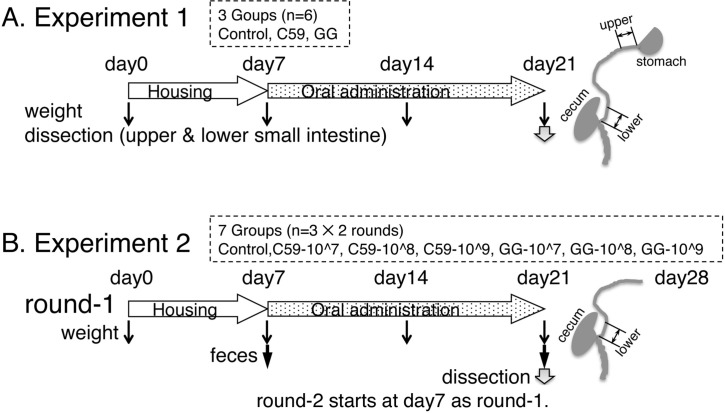
Outline of the animal experiments. (A) Experiment 1. The mice were divided into three groups (control, C59, and GG groups; *n* = 6 per group) and administered 0.2 ml of PBS (control) or LAB suspensions (C59 and GG groups) for 2 weeks. (B) Experiment 2. The mice were divided into seven groups (control, GG-10^7, GG-10^8, GG-10^9, C59-10^7, C59-10^8, and C59-10^9 groups; *n* = 6 per group per experiment) and was performed in duplicate (round-1 and round-2). Round 2 (n = 3 per group) started a week after the start of the round 1 (n = 3 per group).

Experiment 1 involved three groups of animals (control, GG, and C59 groups; *n* = 6 animals per group) (**Fig 1A**). Mice in the GG and C59 groups received bacterial suspensions [OD_620_ = 10, corresponding to 1 × 10^9^ colony-forming units (CFU) per day] of GG and C59, respectively,. After sacrifice, the upper small intestine (2 cm) and the lower small intestine (the terminal ileum; 2 cm) were excised, and immediately placed in RNAlater (QIAGEN, Valencia, CA). RNA was prepared using an RNeasy kit (QIAGEN).

Experiment 2 involved seven animal groups (control, GG-10^7, GG-10^8, GG-10^9, C59-10^7, C59-10^8, and C59-10^9 groups; *n* = 6 per group per experiment) (**Fig 1B**). Mice in the GG-10^7, GG-10^8, and GG-10^9 groups received GG suspensions corresponding to 1 × 10^7^ CFU/day, 1 × 10^8^ CFU/day, and 1 × 10^9^ CFU/day, respectively. Mice in the C59-10^7, C59-10^8, and C59-10^9 groups received C59 suspensions corresponding to 1 × 10^7^ CFU/day, 1 × 10^8^ CFU/day, and 1 × 10^9^ CFU/day, respectively. The feces of each mouse were collected before administration on the first day and after administration on the last day, and suspended by vortex-mixing to a final concentration of 0.1 g of wet weight/ml in PBS containing 1% fetal calf serum. Fecal extracts were obtained by centrifugation (10,000 × *g*, 10 min) and kept at -80°C until use. At day 15, the lower small intestine was taken, and RNA was purified as described above. The body weight gain during the Experiment 2 was analyzed using the MIXED procedure of SAS (SAS Institute, Cary, NC).

Experiment 3 was performed in the same way as Experiment 1. The small intestine was removed and intestinal fluids, obtained by flushing the small intestine with 10 ml PBS, were kept at -80°C until use.

Experiment 4 was performed in the same way as Experiment 1 with two groups of mice (control and GG). The small intestine was removed and used to isolate lymphoid follicles (described below).

### Microarray analysis

The GeneChip mouse 430 2.0 array; one-cycle target labeling and control reagents kit; and hybridization, wash, and stain kit were purchased from Affymetrix (Santa Clara, CA). Total RNA (5 μg) from the upper and lower intestine samples from five randomly selected mice was processed to prepare biotin-labeled complementary RNA using one-cycle target labeling and control reagents kit according to the manufacturer’s instructions. Biotin-labeled cRNA was fragmented and used as a probe for hybridization to the GeneChip mouse 430 2.0 array. Fluorescence data were scanned by a GeneChip Scanner controlled by the GeneChip operating software (GCOS) 1.2 (Affymetrix).

### DNA microarray data analysis

Affymetrix GCOS software was used to convert the array images to numerical values indicating the intensity of each probe (CEL files). The microarray data were analyzed using the statistical language R (http://www.r-project.org/) and Bioconductor (http://www.bioconductor.org/). The annotation file for the mouse 430 2.0 array was downloaded from Affymetrix (http://www.affymetrix.com/). The CEL file data were quantified using robust multi-array analysis (RMA) [39]. To identify differentially expressed genes (DEGs), the rank products method [40] was used (number of permutations = 1000, cut-off = 0.05). Probe sets with a false discovery rate (FDR) <0.05 were considered to indicate DEGs. Principal component analysis (PCA) was performed using the R function ‘prcomp’. Principal component scores of experimental groups were compared using ANOVA and Tukey's test. The component loadings of PC1 and PC3 of each gene were plotted to detect genes contributing to PC1 and PC3. The gene list enrichment analysis was performed using PANTHER v. 11.1 (Protein ANalysis THrough Evolotionary Relationships, http://pantherdb.org) [41]. The lists of up-regulated or down-regulated genes (FDR < 0.05) were analyzed by a PANTHER over-representation test (released July 15, 2016) with the Gene Ontology (GO) database (released December 28, 2016). We considered GO terms that had 30–300 genes [42]. All microarray data were submitted to the National Center for Biotechnology Information (NCBI) Gene Expression Omnibus (http://www.ncbi.nlm.nih.gov/geo/): dataset GSE72804 for the upper and lower small intestine data, and GSE84949 for the dose effect data.

### Enzyme-linked immunosorbent assay (ELISA)

Total IgA concentration in the fecal extracts or intestinal fluids was determined using a mouse IgA enzyme-linked immunosorbent assay (ELISA) quantitation set (Bethyl Laboratories, Inc., Montgomery, AL). Protein concentrations of intestinal fluids were determined by the BCA protein assay kit (Pierce). The concentration of IgA before and after the oral administration of LAB was analyzed using the MIXED procedure of SAS (SAS Institute). The effect of LAB treatment (LAB-treat; control vs. six LAB administration groups), days (Days; before and after administration), and the interaction of LAB-treat and Days parameters were analyzed. The difference of the concentration of IgA in intestinal fluids between the control and the sCSDS groups (Experiment 3) was analyzed by ANOVA. Because the difference of the concentration in IgA was not significant, a post hot test was not performed.

### Immunostaining of the isolated lymphoid follicles (ILFs) in the small intestine

The small intestines of mice in Experiment 4 were analyzed. The immunostaining detection of isolated lymphoid follicles (ILFs) was performed according to the method of McDonald and Newberry [43], with some modifications. The ILF immunostaining protocol has been deposited in protocols.io (dx.doi.org/10.17504/protocols.io.jr3cm8n). The CXCR5-positive ILFs in each segment were independently counted under a stereomicroscope by three investigators, and the number of CXCR5-positive ILFs were averaged in each segment. The numbers of ILFs in control groups and the GG-administered group were statistically compared using Student’s t-test.

## Results and discussion

### The effect of LAB administration on gene expression in the upper and lower regions of the small intestine

In Experiment 1, gene expression in the upper (including the duodenum) and lower (the terminal ileum) small intestine was compared to identify the region of the small intestine that is affected by LAB strain administration (**Fig 1A**). After 2 weeks of the administration period, gene expression in the upper and lower small intestine was analyzed using the GeneChip mouse 430 2.0 array with 45,101 native features. We applied three quantification methods (RMA [39], DFW [44], and qFARMS [45]) to the raw data. Hierarchical cluster analysis revealed that three methods did not provide good segregation when expression data of 45,101 probes were used, suggesting that administration of a strain of LAB did not affect overall gene expression (**S1 Fig**). Microarray data from the three animal groups were quantified using RMA followed by collapsing features into gene symbols and 21,815 genes were further analyzed.

Rank products analysis [40] was more sensitive in detecting DEGs (FDR < 0.05) in the control group and groups administered LAB (**S1–S4 Tables**). The number of DEGs in the upper small intestine and terminal intestine of each mouse administered C59 and GG were shown in **Table 1**. Total number of DEGs was higher in the terminal ileum than in the upper small intestine of both C59- and GG-administered mice, suggesting that the terminal ileum is more suitable for investigating the effects of administration of LAB strains.

**Table 1:** Number of differentially expressed genes (DEGs, FDR < 0.05)) in upper small instestine and terminal ileum of mice administered C59 and GG.

small intestine	upper small intestine			terminal ileum		
administered LAB	C59	GG	common DEGs^a^	C59	GG	common DEGs^a^
up-regulated	94	83	(37)	132	95	(30)
						(5)^b^
down-regulated	47	89	(32)	172	142	(43)
			(24)^c^			(4)^c^
total	141	172		304	237	

^a^ Number of common DEGs represented in () is included in respective DEGs in C59 and GG.

^b^ DEGs up-regulated in GG-administered mice and down-regulated in C59-administered mice.

^c^ DEGs up-regulated in C59-administered mice and down-regulated in GG-administered mice.

To detect biological processes defined by GO that were affected by the administration of LAB, gene list enrichment analysis using the PANTHER over-representation test was employed, using lists of up-regulated or down-regulated genes (FDR < 0.05) in each LAB-administered group (**Table 2**). In the upper small intestine, the administration of C59 down-regulated the same biological processes as the administration of GG, suggesting that the effects were LAB-specific but not strain-specific. *Fga*, *Fgb*, and *Fgg* encoding fibrinogen subunits, and *Plg* encoding plasminogen, were significantly down-regulated (FDR<0.05) in groups administered both C59 and GG, among the genes associated with the biological processes “plasminogen activation”, “positive regulation of heterotypic cell-cell adhesion”, and “fibrinolysis”. These genes were not detected as DEGs in the terminal ileum. This observation raised the possibility that down-regulation of fibrinogen subunit and plasminogen gene expression in the upper small intestine might be a general effect of LAB administration. The oral administration of *Lactobacillus casei* CRL431 was shown to activate effectively coagulation and inhibit fibrinolysis during a pneumococcal infection; this was mediated by an induction of high levels of IL-4 and IL-10 [46]. In the current study, the oral administration of C59 or GG did not significantly affect the expression of *Il4* and *Il10* in either the upper or lower small intestine in a healthy mouse. Whether administration of LAB affects blood coagulation itself by down regulating of expression of coagulation-related genes remains to be investigated. With respect to *Plg* gene expression in the upper small intestine, although it has been shown that the cell surface components of C59 can activate plasminogen *in vitro* [38], the effect of oral administration of C59 could be distinct from the *in vitro* mechanism.

**Table 2:** PANTHER Overrepresentation Test of gene expression in upper small intestine and terminal ileum of mice administered C59 and GG.

Intestine	LAB	up/ down () *	GO biological process	Ref-list (22322)	loaded	expected	Fold enrichment	P-value
upper small instestine	C59	up	digestion (GO:0007586)	94	10	0.37	27.0	4.69E-08
		(94)	carbohydrate transport (GO:0008643)	78	6	0.31	19.5	6.17E-03
			steroid metabolic process (GO:0008202)	197	9	0.78	11.6	7.92E-04
			response to nutrient levels (GO:0031667)	238	9	0.94	9.6	3.81E-03
		down (47)	plasminogen activation (GO:0031639)	10	4	0.02	> 100	2.35E-05
			positive regulation of heterotypic cell-cell adhesion (GO:0034116)	10	3	0.02	> 100	5.99E-03
			fibrinolysis (GO:0042730)	15	4	0.03	> 100	1.18E-04
	GG	up (83)	n.d.					
		down (89)	positive regulation of heterotypic cell-cell adhesion (GO:0034116)	10	3	0.03	97.1	3.68E-02
			plasminogen activation (GO:0031639)	10	3	0.03	97.1	3.68E-02
			fibrinolysis (GO:0042730)	15	4	0.05	86.3	1.36E-03
terminal ileum	C59	up (132)	n.d.					
		down (176)	n.d.					
	GG	up (95)	brown fat cell differentiation (GO:0050873)	34	5	0.13	37.7	2.18E-03
			lymph node development (GO:0048535)	28	4	0.11	36.7	4.05E-02
			B cell receptor signaling pathway (GO:0050853)	52	5	0.2	24.7	1.73E-02
			regulation of leukocyte chemotaxis (GO:0002688)	101	6	0.39	15.2	2.53E-02
			B cell activation (GO:0042113)	155	8	0.6	13.2	1.55E-03
			cell chemotaxis (GO:0060326)	165	7	0.64	10.9	3.36E-02
			regulation of leukocyte migration (GO:0002685)	165	7	0.64	10.9	3.36E-02
			adaptive immune response (GO:0002250)	274	9	1.07	8.4	1.09E-02
		down (143)	vasodilation (GO:0042311)	34	5	0.12	42.1	1.26E-03
			regulation of lipid metabolic process (GO:0019216)	298	9	1.04	8.6	8.56E-03

* number in () indicates the number of analyzed genes (FDR<0.05).

By contrast to the upper small intestine, common biological processes affected by the oral administration of C59 or GG were not identified in the terminal ileum and only administration of GG affected several biological processes, suggesting a GG-specific effect in that intestinal segment. Significant (*p* < 0.05) enrichment of B cell-related GO terms was observed among up-regulated genes in the group administered GG. The following DEGs were associated with “brown fat cell differentiation”: *Adb3*, *Adig*, *Adipoq*, *Fabp4*, and *Scd1*. The following DEGs associated with “lymph node development”, “B cell receptor signaling pathway”, “B cell activation”, “cell chemotaxis”, and “adaptive immune response” were identified: *Arhgef16*, *Blk*, *Ccr6*, *Ccr7*, *Cd79a*, *Cd79b*, *Cr2*, *Cxcr13*, *Cxcr5*, *Fcamr*, *Ighg*, *Klhl6*, *Ltb*, *Ms4a1*, *Pdgfra*, *Saa2*, *Saa3*, and *Tnfrsf13c*. These results suggest that GG administration exerts a more pronounced effect on the activation of immune cells than C59 administration. Pronounced immunostimulatory effects of GG have been reported in various studies [16, 17, 29–31, 47]. Our results confirmed these immunostimulatory effects, as reflected by the host’s intestinal gene expression. Since more DEGs were detected in the terminal ileum and GG-specific enrichment was observed when the terminal ileum was examined, we decided to focus on the terminal ileum in subsequent analyses.

### The effect of strain and dose of orally administered LAB on the gene expression in the terminal ileum

In Experiment 1, we showed that oral administration of 10^9^ CFU/day of C59 or GG for 2 weeks significantly changed intestinal gene expression. To evaluate the strain-specific and dose-related effects of LAB on gene expression in the terminal ileum, 1 × 10^7^, 1 × 10^8^, and 1 × 10^9^ CFU/day of strains C59 and GG were administered in Experiment 2 (**Fig 1B**). Throughout the experiment, the administration of various doses of LAB had no effect on animal body weight gain. The DEGs between the control group and each LAB administered group were identified using rank products analysis (**Table 3**, **S5–S10 Tables**) [40]. Among up- and down-regulated DEGs, number of common DEGs were also counted in C59- and GG- administered groups, respectively (Table 3). 134 up-regulated DEGs and 6 down-regulated DEGs were found in all LAB-administered groups. To clarify the reproduction of DEGs in Experiment 1 (Table 1) and Experiment 2 (Table 3), up- and down-regulated DEGs were compared. In the case of C59-administered group, 31 genes (23%) out of 132 up-regulated DEGs in Experiment 1 were reproduced in Experiment 2 (C59-10^9 group), whereas only 15 genes (8.7%) out of 172 down-regulated DEGs were reproduced. In the case of GG-administered group, 36 genes (38%) out of 95 up-regulated DEGs and 9 genes (6%) in 142 down-regulated DEGs in Experiment 1 were reproduced in Experiment 2 (Table 3, GG-10^9). Although C59-10^9 and GG-10^9 groups showed the highest number of DEGs, no clear dose-dependent effects on the number of DEGs were observed.

**Table 3:** Number of differentially expressed genes (DEGs, FDR < 0.05) in the terminal ileum of mice administered different doses of C59 and GG.

administered LAB	C59				GG			
dose group	10^7	10^8	10^9	common DEGs a (C59)	10^7	10^8	10^9	common DEGs a (GG)
up-regulated	254	250	292	(157)	558	465	636	(254)
down-regulated	61	87	59	(10)	138	140	242	(63)
total	315	337	351	(167)	696	605	878	(317)

a Number of common DEGs in () is included in respective DEGs.

**Fig 2:**
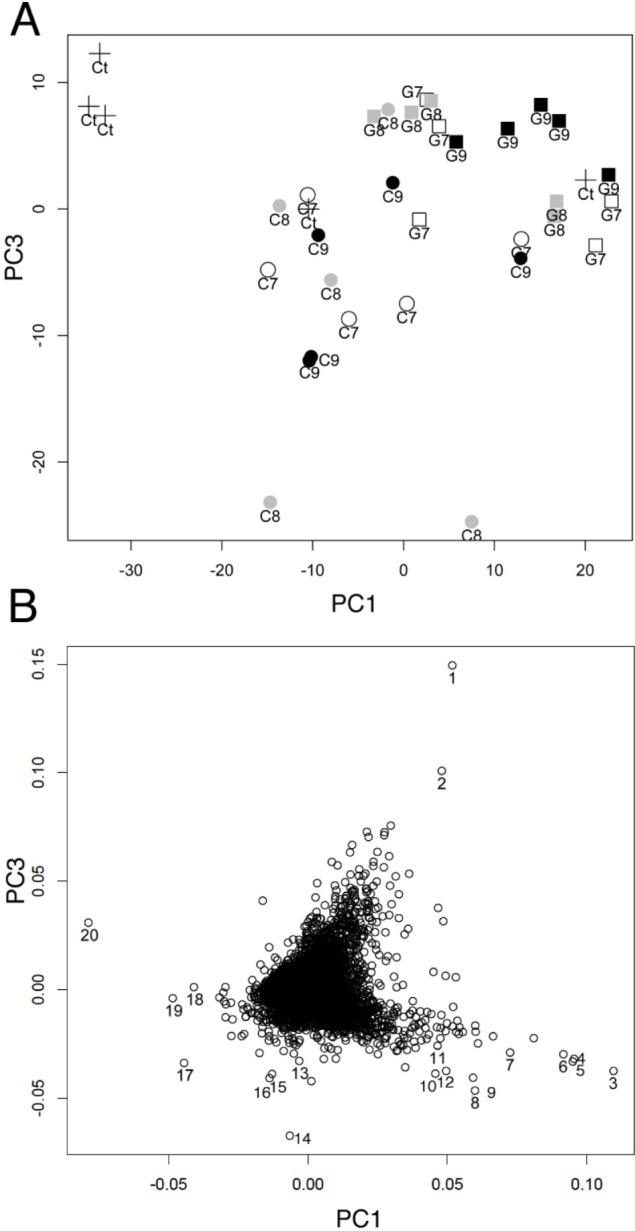
Principal component analysis of gene expression of the terminal ileum after the administration of various doses of C59 and GG. A. PCA plot using PC1 and PC3 gene expression data quantified by RMA. Each dot indicates control (Δ), C59-10^7 (○), C59-10^8 (⊗), C59-10^9 (●), GG-10^7 (□), GG-10^8 (⊠), and GG-10^9 (■) (n = 5 in each group). B, Plot of component loadings of PC1 and PC3 for all genes. Gene with high PC values were numbered: 1, *Hist1h2ao/Hist1h2ap*; 2, *C430003N24Rik*; 3, *Ms4a1*; 4, *Glycam*; 5, *Faim3*; 6, *Ighv14-2*; 7, *Cr2*,; 8, *Serpina1a*; 9, *P2ry10*; 10. *Mndal/Ifi205/Ifi204/Ifi211*; 11, *Ighg/Ighg2b*; 12, *Hmgb1l/Hmgb1*; 13, *Arfgef3*; 14, *Igkv6-20*; 15, *2810043O03Rik*; 16, *B830007D08Rik*; 17, *Slc6a14*; 18, *Retnlb*; 19. *Saa1*; and 20, *Car1*.

To identify the factors contributing to changes in gene expression in the terminal ileum, principal component (PC) analysis (PCA) was performed using array data quantified by RMA (**Fig 2A**). Each gene had a loading in each principal component. PCA was used to determine whether gene expression would be affected by different doses of LAB. PCA of all RMA data (7 groups, n = 5) revealed that the cumulative proportion of top 15 PCs was 0.85. PC1 significantly contributed to the classification between control and GG-10^7 (*p <* 0.05), GG-10^8 (*p* = 0.05), and GG-10^9 (*p <* 0.01), respectively, suggesting that administration of more than 1 × 10^7^ CFU/day of GG changed the gene expression of the host ileum. There was no significant difference between groups at different doses of GG. The PCA plot showed that 14 out of 15 GG groups had a positive PC1 coefficient value (**Fig 2A**). Conversely, PC3 significantly contributed to the classification between the control and the C59-10^8 group (*p <* 0.01). Eleven out of 15 C59 administered groups had a negative coefficient value on PC3. The component loadings of PC1 and PC3 for each gene were plotted to detect the contribution to these two PCs (**Fig 2B**). Genes with higher component loadings on PC1 and lower component loadings on PC3 were identified (**S11 Table**). Interestingly, genes with a high PC1 coefficient were found in DEGs identified in B cell-related GO terms in Experiment 1. Thus, the expression of these genes, *Ccr6*, *Cd79a*, *Cd79b*, *Cr2*, *Cxcr5*, *Klhl6*, *Ms4a1*, *Tnfrsf13c*, would characterize the effect of GG administration.

The result of PCA suggested that the administration of C59 and GG differentially affected the gene expression in the terminal ileum.

In the case of GG administration, a difference in gene expression was observed in the GG-10^7 group. We previously reported that a 7 day consecutive administration of GG (4 × 10^8^ CFU/0.2 ml of PBS) to mice resulted in a marked increase in *Tlr3* gene expression in the small intestine, but did not alter the expression of genes encoding other TLRs [34]. In the current study, a significant up-regulation of *Tlr3* expression was observed in the groups administered both GG and C59 (FDR < 0.05), except for the GG-10^9 group (FDR = 0.05). This was consistent with the observation that LAB strains harbor more dsRNA than other examined pathogenic bacteria [35]. In experiments that employ bone marrow-derived dendritic cells, LAB induces IFN-β production via dsRNA [35]. Changes in the expression of the *Ifnb1* gene encoding IFN-β were not observed in the current study. TLR3 plays a key role in the innate immunity [35, 48], and LAB administration would stimulate the host’s immune responses. The type of dsRNA that is involved in the up-regulation of *Tlr3* and the question of whether LAB dsRNA is involved in the strain-specific probiotic effect should be investigated further.

To detect the dose-dependent effect of LAB administration on the biological processes in the terminal ileum, the PANTHER over-representation test was employed, using lists of genes up-regulated or down-regulated (FDR < 0.05) in each group administered LAB (**S5–S10 Tables**). Although neither the number of DEGs (**Table 3**) nor PCA (**Fig 2**) revealed dose-related effects of LAB, gene list enrichment analysis revealed strain- and dose-related effects on GO biological processes (**Table 4**). Significant enrichment of genes involved in “intestinal absorption” (GO:0050892) was observed in both the C59-10^8 group (*p* = 0.0014) and the C59-10^9 group (*p* = 0.0050); in these groups, six genes, namely, *Abcb1a*, *Aco1*, *Cd36*, *Fabp1*, *Lep*, and *Slc5a1*, were significantly up-regulated (FDR < 0.05).

**Table 4:** PANTHER Overrepresentation Test of gene expression in terminal ileum of mice administered GG and C59.

Group	up/down () *	GO biological process	Ref-list (22322)	loaded	expected	Fold enrichment	P-value
C59 10^7	up (255)	n.d.					
	down (1)	n.d.					
C59 10^8	up (251)	intestinal absorption (GO:0050892)	26	6	0.24	25.5	0.0014
		→digestive system process (GO:0022600)	58	7	0.52	13.34	0.0101
	down (88)	regulation of plasma lipoprotein particle levels (GO:0097006)	43	4	0.07	56.12	0.0069
C59 10^9	up (293)	intestinal absorption (GO:0050892)	26	6	0.29	20.6	0.0050
		steroid metabolic process (GO:0008202)	197	12	2.21	5.44	0.0245
		glycerolipid metabolic process (GO:0046486)	250	15	2.8	5.36	0.0017
	down (59)	n.d.					
GG 10^7	up (559)	regulation of energy homeostasis (GO:2000505)	21	6	0.42	14.27	0.0416
		triglyceride metabolic process (GO:0006641)	62	10	1.24	8.05	0.0058
		regulation of lipid metabolic process (GO:0019216)	298	20	5.97	3.35	0.0309
	down (138)	n.d.					
GG 10^8	up (435)	lipid modification (GO:0030258)	153	13	2.59	5.02	0.0244
		glycerolipid metabolic process (GO:0046486)	250	18	4.23	4.25	0.0035
	down (141)	n.d.					
GG 10^9	up (637)	B cell receptor signaling pathway (GO:0050853)	52	9	1.09	8.22	0.0176
		→immune response-regulating cell surface receptor signaling pathway (GO:0002768)	126	14	2.65	5.28	0.0059
		regulation of B cell proliferation (GO:0030888)	64	11	1.35	8.16	0.0014
		→regulation of lymphocyte proliferation (GO:0050670)	208	17	4.38	3.88	0.0252
		positive regulation of adaptive immune response based on somatic recombination of immune receptors built from immunoglobulin superfamily domains (GO:0002824)	91	11	1.92	5.74	0.0412
		B cell activation (GO:0042113)	155	17	3.26	5.21	0.0005
		positive regulation of cell activation (GO:0050867)	294	21	6.19	3.39	0.0149
	down (242)	n.d.					

* number in () indicates the number of analyzed genes (FDR<0.05).

Specific GO term enrichment was not apparent for down-regulated genes in GG animals. Similar to the results of Experiment 1 (**Table 1**), enrichment of “B cell receptor signaling pathway” and “B cell activation” processes was also observed in the GG-10^9 group in Experiment 2. These results suggested that GG cells interact specific intestinal immune cells to activate B cells and administration of 1 × 10^9^ CFU/day of GG cells is required to execute this activity. The following 33 genes listed under “B cell receptor signaling pathway” and “B cell activation” were up-regulated in GG-10^9 group: *Aicda*, *Bank1*, *Blk*, *Btk*, *Btla*, *Ccr6*, *Cd19*, *Cd24a*, *Cd40*, *Cd79a*, *Cd79b*, *Cr2*, *Cxcr5*, *Fnip1*, *Gcsam*, *Hhex*, *Ighg*, *Ighg1*, *Ighg3*, *Ighv14-2*, *Il2rg*, *Klhl6*, *Lyl1*, *Malt1*, *Mef2c*, *Ms4a1*, *Pawr*, *Ptprc*, *Sash3*, *Stap1*, *Tfrc*, *Tnfrsf13b*, and *Tnfrsf13c*. Such enrichment was not observed in either the GG-10^7 or the GG-10^8 group, indicating that administration of 1 × 10^9^ CFU/day of GG increased the expression of B-cell related genes. Among B-cell related genes, *Aicda*, *Bank1*, *Btla*, *Ccr6*, *Cd19*, *Cd79a*, *Cd79b*, *Cr2*, *Cxcr5*, *Ighv14-2*, *Klhl6*, *Ms4a1*, *Stap1*, *and Tnfrsf13c* were also among the top 50 genes with high PC1 loading (**S11 Table**). Interestingly, the expression of these genes decreased with an increasing dose of administered C59, while it increased with an increasing dose of administered GG. Specifically, the expression of *Cd79b*, *Cxcr5*, *Aicda*, and *Ccr6* genes was down-regulated in the C59-10^8 and C59-10^9 groups. Concerning the adherence to the intestinal epithelial cells, GG is well known to adhere to the intestinal mucus via the pili [12], whereas C59 is quickly excreted with the feces, 6 h after oral administration [28]. The ability of GG to persist in the intestinal tract longer than C59 may explain its greater immunostimulatory effect. However, the observed opposite effect on gene expression cannot be explained by the strain’s adherence ability. Strain C59 weakly stimulates macrophages, inducing the production of low levels of such inflammatory cytokines, such as IL-12 [28, 49]. In addition, oral administration of live C59 suppressed IgE antibody production in OVA-sensitized mice [28]. This suggests that the difference in the effect of C59 and GG on murine gene expression is not associated with the difference between *Lactobacillus* and *Lactococcus*, but is strain-specific.

We previously observed a significant down-regulation (*p* < 0.001) of genes involved in the immune response in the terminal ileum in mice exposed to sub-chronic and mild social defeat stress, a murine model of depression [50]. Because GG can stimulate B cell-related pathways in the terminal ileum, intake of GG may attenuate stress-induced symptoms, including perturbation of the intestinal microbiota and metabolites, by stimulating the immune responses.

### The effect of LAB administration on the amount of secretory IgA in feces

**Fig 3:**
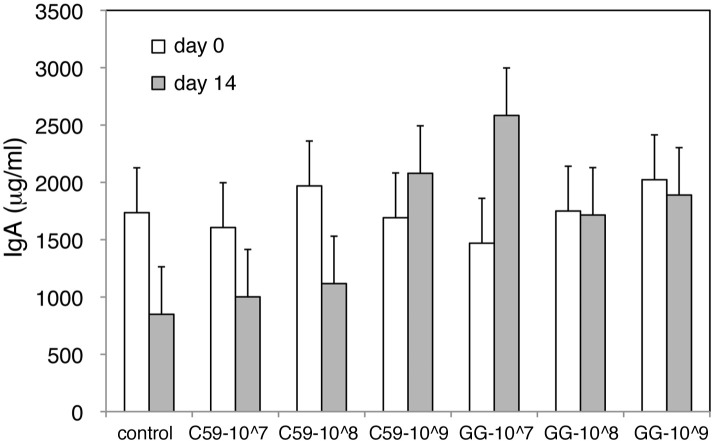
Fecal concentration of secretory IgA before and after LAB administration. The fecal concentration of secretory IgA before and after administration of C59 or GG during experiment 2 was determined using ELISA. The white and gray bars represent the concentration of secretory IgA (μg/ml) before and after LAB administration, respectively. The data are shown as the least-squares means ± SE (n = 5 in each group).

To investigate the effect of oral C59 and GG administration on the mucosal immune system, fecal secretory IgA levels were compared before and after a 15-day administration of these LAB strains. Statistical analysis (the MIXED procedure) showed that the effects of LAB-treat (control, C59, GG) and Days (day 1, day 15) were not significant. No statistically significant interaction between LAB-treat and Days was observed (*P* = 0.13). The levels of secretory IgA before and after LAB administration are shown in **Fig 3**. Concentrations of secretory IgA were also examined in intestinal fluids. The concentrations in control, C59 and GG groups were 1.17 ± 0.42, 1.12 ± 0.60 and 1.34 ± 0.74 (μg/μg protein), respectively, demonstrating that there was no significant difference (p = 0.94) between the three groups.

Viable GG cells stimulate rotavirus-specific IgA responses in patients with rotavirus diarrhea [51], while non-viable GG stains enhance the systemic and mucosal immune responses, resulting in the elevation of OVA-specific secretory IgA levels in OVA-sensitized mouse [52]. In our experiments, the fecal secretory IgA levels in non-sensitized mice were not significantly affected by a 2 week administration of GG. Other than GG, Carasi et al. investigated the induction of IgA levels after a 14 d administration of *Lactobacillus kefiri*; the IgA levels were further increased after 21 d administration [53]. Oral administration of *Lactobacillus plantarum* strain AYA for 28 d enhances IgA secretion and increases survival rates following influenza virus infection in mouse [54]. The duration and dose of LAB administration required to observe enhanced IgA secretion are strain-dependent and critical for understanding immune responses to LAB.

### The effect of LAB administration on CXCR5-positive ILFs

The mucosal immune system of the intestine comprises the intestinal lamina propria cells and the intraepithelial lymphocytes, in addition to such organized lymphoid structures as the Peyer’s patches (PPs) and ILFs. Unlike the programmed development of PPs, the maturation of ILFs from cryptopatches requires induction by the intestinal microbiota [55]. The chemokines CCL20 and CXCL13, and their respective receptors CCR6 and CXCR5, play a role in the development of ILFs [56–58]. The expression of *Ccl20* and *Cxcl13*, and their respective receptor genes (*Ccr6* and *Cxcr5*), in the ILFs of aged mice were 3–4 fold lower than those of young mice [56]. In the current study, compared with control group, the expression of the *Cxcl13* (ranked 32 among the up-regulated DEGs, FDR < 0.001), *Cxcr5* (ranked 57 among the up-regulated DEGs, FDR < 0.001), and *Ccr6* genes (ranked 94 among the up-regulated DEGs, FDR < 0.001) was significantly increased in the GG-10^9 group; the expression of *Ccl20* was not significantly changed.

**Fig 4:**
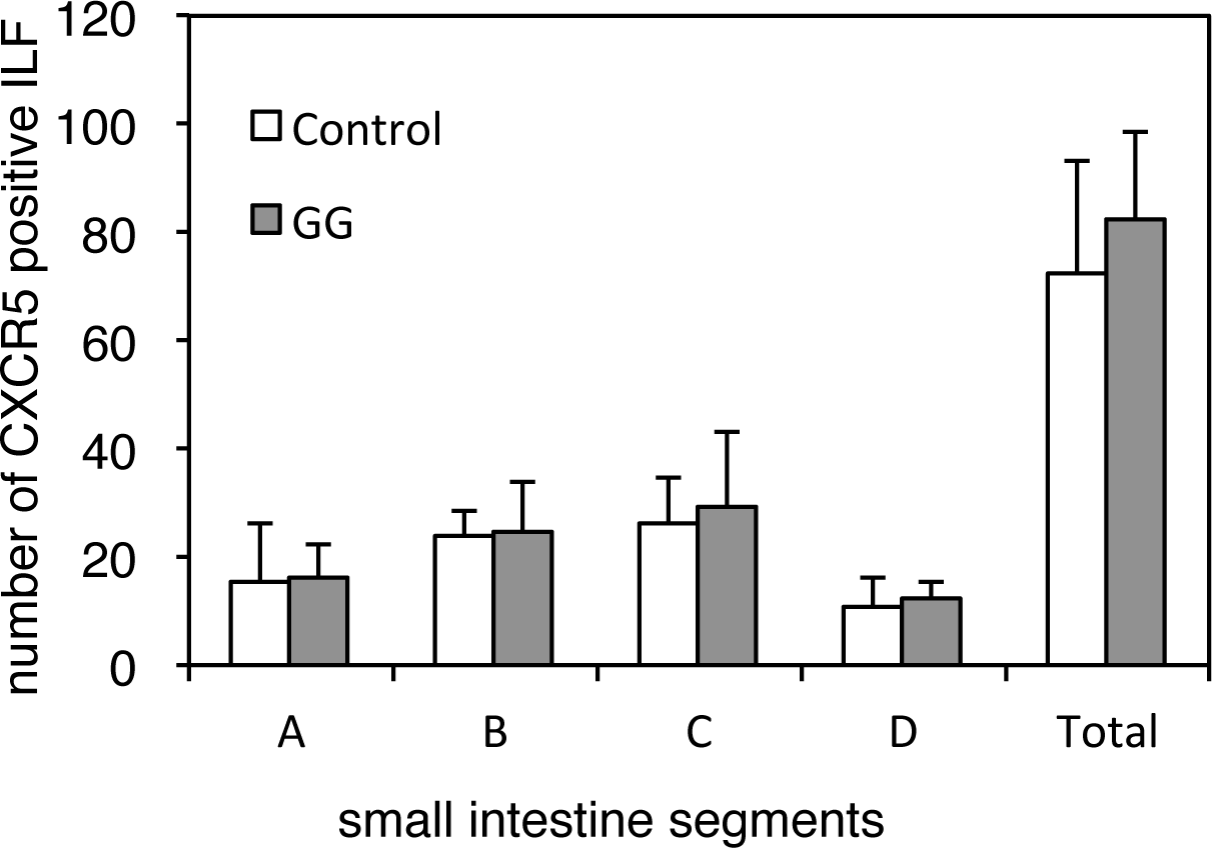
The effect of GG administration on the number of CXCR5-positive ILFs in the murine small intestine. After 2 weeks of GG administration (1 × 10^9^ CFU/day) or PBS (n = 5 in each group), the small intestines were divided into four equal segments, from proximal to distal (A–D), labeled with anti-CXCR5 antibody and HRP-conjugated anti-rabbit antibody, and visualized with DAB. The CXCR5-positive ILFs in each intestinal fragment were counted under a stereomicroscope.

We also examined the effect of oral GG administration on ILF development. After 2 weeks of GG administration (1 × 10^9^ CFU/day), the small intestines were stained with anti-CXCR5 antibody, and CXCR5-positive ILFs were counted. No significant differences in the number of CXCR5-positive ILFs in the control group and mice administered GG were observed (**Fig 4**). The terminal ileum, evaluated by the microarray analysis, was located in segment D, where the number of ILFs was lower than in other segments. The number of ILFs was not significantly affected by GG administration. Further studies are required to identify the immune cells recognizing individual GG cells and the mechanism through which the mucosal immune system is up-regulated by GG administration.

## Conclusions

In the current study, the effect of oral LAB administration on gene expression in the small intestine was investigated using a conventional mouse model. Intestinal site-specific, strain-specific, and dose-specific gene expression was observed. The oral administration of strains C59 and GG significantly (FDR < 0.05) down-regulated the expression of genes encoding fibrinogen subunits and plasminogen in the upper small intestine. PCA revealed that oral administration of GG changed gene expression of the terminal ileum at a dose of at least 1 × 10^7^ CFU/day for 2 weeks. Although the hierarchical cluster analysis showed that administration of LAB did not affect overall gene expression, gene list enrichment analysis suggested that distinct strains would interact with specific intestinal cells and differentially affect their gene expression as reflected by various GO biological processes. Genes associated with the B cell receptor signaling pathway and B cell activation were up-regulated in the terminal ileum following GG administration at a dose of 1 × 10^7^ CFU/day, in agreement with the reported immunostimulatory effects of GG. The distinct effects of GG and C59 on gene expression in the small intestine will provide useful information about intestinal responses of healthy animals to probiotic strains and clues to understanding the strain-specific mechanisms of probiotic action.

## Supporting information

S1 Fig Comparison of the hierarchical clustering dendrograms obtained by the three data-quantification methods. Raw data from the upper small intestine and the terminal ileum were quantified by qFARM, DFW, and RMA, as indicated. Left panels are data from the upper small intestine and right panels are those from the terminal ileum. Each sample name comprises the strain name (cont, control; C59, *L*. *lactis* C59; GG, *L*. *rhamonosus* GG) and the number corresponding to the individual mouse. The vertical scale represents the distance between clusters.
(EPS)

S1 Table DEGs of upper ileum administered C59 (FDR<0.05). (XLSX)

S2 Table DEGs of upper ileum administered GG (FDR<0.05). (XLSX)

S3 Table DEGs of terminal ileum administered C59 (FDR<0.05). (XLSX)

S4 Table DEGs of terminal ileum administered GG (FDR<0.05). (XLSX)

S5 Table DEGs of terminal ileum of C59-10^7 group (FDR<0.05). (XLSX)

S6 Table DEGs of terminal ileum of C59-10^8 group (FDR<0.05). (XLSX)

S7 Table DEGs of terminal ileum of C59-10^9 group (FDR<0.05). (XLSX)

S8 Table DEGs of terminal ileum of GG-10^7 group (FDR<0.05). (XLSX)

S9 Table DEGs of terminal ileum of GG-10^8 group (FDR<0.05). (XLSX)

S10 Table DEGs of terminal ileum of GG-10^9 group (FDR<0.05). (XLSX)

S11 Table Top 50 genes with higher and lower PC1 and PC3 component loadings. (XLSX)
